# Primary Immunodeficiency Diseases and Gastrointestinal Distress: Coping Strategies and Dietary Experiences to Relieve Symptoms

**DOI:** 10.1177/1049732320967908

**Published:** 2020-11-04

**Authors:** Katrine K. Brede, Margareta Wandel, Ingrid Wiig, Charlotte von der Lippe

**Affiliations:** 1 University of Oslo, Oslo, Norway; 2 Oslo University Hospital HF, Oslo, Norway

**Keywords:** primary immune deficiency, gastrointestinal distress, qualitative study, coping strategies, qualitative, thematic analysis, Norway

## Abstract

In this article, we focus on adults with primary immunodeficiency disease (PID) and their experiences with gastrointestinal (GI) distress with the aim of exploring how they experience living with their condition and the actions they take to relieve GI distress. Twelve adults with PID and GI distress participated in semi-structured, in-depth interviews. The interviews were analyzed following the steps of thematic analysis (TA). The study revealed the complexity of the psychosocial aspects of living with PID and GI distress. Participants experienced GI distress to be highly challenging in daily life and felt they had to cope with the condition alone, without adequate help from the health care service. Participants used a wide and diverse range of coping strategies, and the search for normalcy was evident. Health care professionals should be more proactive in supporting individuals with PID in their struggle to find solutions to problems arising from GI distress.

## Introduction

Primary immunodeficiency diseases (PIDs) result from internal defects of the immune system of a genetic and often inherited origin (Wood et al., 2007). Examples of such defects are selective immunoglobulin (Ig) A deficiency, common variable immunodeficiency (CVID), X-linked agammaglobulinemia (XLA), and chronic neutropenia (CN). Increased susceptibility to infections, autoimmunity, lymph proliferation, granulomatous process, atopy, and malignancy characterize PIDs (Raje & Dinakar, 2015). Atypical and opportunistic organisms can cause infections. The infections usually have a longer duration and a more serious course than normal, and they may require more intensive treatment. The overall clinical picture depends on the specific type of underlying immune defect.

The prevalence of PIDs varies depending on the subgroup of immunodeficiency. Selective IgA deficiency has the highest frequency, but most individuals with IgA deficiency are asymptomatic and are usually not included in surveys (Chapel & Cunningham-Rundles, 2009). A Norwegian epidemiological study from 2000 found a prevalence of PIDs of 6.82 per 100,000 inhabitants (one in 14,663) (Stray-Pedersen et al., 2000). Primary antibody deficiencies, mainly CVID, represented 50.8% of these PIDs (one in 28,902). Estimates from the United States state a population prevalence of PIDs of approximately one in 1,200 persons (Boyle & Buckley, 2007).

PIDs are chronic diseases requiring lifelong treatment. Replacement therapy with human immunoglobulin is the mainstay treatment in addition to antibiotics for the treatment and prevention of infections, and appropriate therapy for noninfectious complications (Berger, 2008; Yong et al., 2011). Replacement therapy, supplied through hospital-based intravenous immunoglobulin (IVIG) infusions or home-based subcutaneous immunoglobulin (SCIG) infusions, can prevent or alleviate infections and improve physical functioning. Stem cell transplants and bone marrow transplants are associated with risk and are therefore used only in the most severe cases (Bonilla et al., 2015). Gene therapy has been successful in some types of PIDs (Bonilla et al., 2015). Lifestyle changes to prevent infections, like washing their hands and keeping distance from people with contagious diseases, can significantly improve morbidity (Raje & Dinakar, 2015). A healthy diet is important to provide normal growth and development, as well as reducing oxidative stress and repairing damaged cells. A lack of adequate nutrition can increase vulnerability to infections. Physical activity is also important for individuals with PIDs to ensure optimal health (Raje & Dinakar, 2015).

Individuals with PIDs frequently present with pathological conditions in the gastrointestinal (GI) tract (Lai Ping So & Mayer, 1997), such as chronic or acute diarrhea, malabsorption, and GI pain (Kobrynski & Mayer, 2011). GI disorders are the second most common complication in PIDs after sinopulmonary disease (Lai Ping So & Mayer, 1997).

The incidence of GI manifestations among individuals with CVID is high (20%–60%) and can be the initial manifestation of the disease (Atalaia-Martins et al., 2017; Lai Ping So & Mayer, 1997).

Noninfectious GI pathology is widespread, including nodular lymphoid hyperplasia, atrophic gastritis, lymphocytic colitis, and pathology-mimicking diseases like celiac disease and inflammatory bowel disease (Agarwal & Mayer, 2009; Bonilla et al., 2016).

Despite increased blood concentrations of dietary antigens in hypogammaglobulinemia, there is little evidence to support an increased prevalence of food sensitivity or allergy (Lai Ping So & Mayer, 1997). As of today, dietary therapy for GI distress in individuals with PID is empirical and largely based on either individuals’ or health care professionals’ experiences.

Living with a chronic disease has consequences for daily life (Martz & Livneh, 2007; Robinson, 2017). Individuals with PID need to handle symptoms, treatment, complications, such as GI distress, and the unpredictability in terms of symptoms and disease progression. To cope with such numerous life challenges, people with chronic illnesses require a wide range of coping strategies (Anderson & Asnani, 2013; Garrino et al., 2015; Martz & Livneh, 2007; Robinson, 2017). Lazarus and Folkman (1984) developed a cognitively oriented theory of stress and coping and define coping as “the cognitive and behavioural efforts to master, reduce or tolerate the internal and/or external demands that are appraised as taxing or exceeding the resources of the person.” (p. 19) Personal and socioecological resources, for example, health and energy, beliefs about control, problem-solving skills, social support, and material resources partly determine the way a person copes (Lazarus & Folkman, 1984).

## The Relevance of Qualitative Research in Clinical Nutrition and Rationale for This Study

Qualitative research methods can gain insights into people’s perspectives and the meanings they give to experiences (Braun & Clarke, 2013). Such methods are therefore well suited for investigating the psychosocial aspects of living with PID and GI distress. Although studies have shown that GI distress among individuals with PID is widespread (Cunningham-Rundles & Bodian, 1999; Daniels et al., 2007; Jørgensen et al., 2016), research on how they experience and deal with these problems is limited. To facilitate the process of self-management of individuals with chronic diseases, health care professionals should tailor various clinical resources to individuals’ needs (Schulman-Green et al., 2012). However, to do so health care professionals must have knowledge about the needs. It is important to explore different experiences people with PID have. Results from this study can be used to inform organizations such as the U.S. Immune Deficiency Foundation who are currently generating data on immunodeficiency disease types, incidence, and treatment (U.S. Immune Deficiency Foundation., 2020: https://primaryimmune.org/living-pi/idf-ephr-and-pi-connect). The aim of this study was to explore coping strategies used by individuals with PID, focusing especially on their awareness of the influence of diet on GI distress.

## Method

### Design

We used an explorative qualitative research approach with in-depth interviews to gather data.

### Recruitment and Participants

Participant recruitment was based on a purposive sampling of adults (over 18 years old, who could read and speak Norwegian) with both PID and GI distress. Exclusion criteria were inflammatory GI diseases not caused by the immunodeficiency. Participants were recruited via invitation letter on the website of the Norwegian Patient Organization for Primary Immunodeficiency and via the staff at a medical day-care unit, at Oslo University Hospital. Twelve adults met the inclusion criteria and completed the interview (Figure 1, Supplemental File).

### Data Collection and Analysis

Katrine K. Brede, who did not have any prior knowledge about PID diagnoses in general, conducted 11 face-to-face interviews and one interview by telephone. The interviews took place between October 2017 and December 2017 and were audiotaped. The time and place for the interviews were set according to the convenience for the participants. A semi-structured interview guide was developed based on a literature review and on discussions with professionals familiar with the diagnosis. The following topics were included: (a) living with PID, (b) eating habits, (c) GI distress, (d) considerations to minimize GI distress, and (e) daily life challenges. Example questions from the interview guide included “Please describe how you experience living with stomach problems” and “Can you tell about any considerations you may take to minimize the problems?”

The analysis was based on an inductive thematic analysis (TA) approach (Virginia Braun & Clarke, 2006). The purpose of TA was to identify patterns of meaning across the interviews (data set) to provide an answer to the research question. The interviews lasted on average 40 minutes (range: 25 minutes–1 hour). Katrine K. Brede transcribed the interviews verbatim. The anonymized transcripts, supported by field notes, constituted the data material. Katrine K. Brede coded the entire material inductively, line by line, in tandem with the co-authors. Subsequently, the authors examined and organized the codes to identify broader patterns of meaning toward answering the research questions. The authors translated illustrative quotes to English and allocated pseudonyms to the participants. Saturation was not a criterion in deciding sample size; however, we do feel saturation was reached since no new themes emerged in the final set of interviews.

### Compliance With Ethical Standards

Ethical approval for the study was obtained from the Regional Committee for Medical Research Ethics (Health Region South-East, reference 2017/1336). The Declaration of Helsinki was followed to ensure the protection of participants’ privacy (Mand et al., 2013). The participants were informed that their participation was voluntary and that they could withdraw from participation at any time. All participants signed a written consent form.

## Results

### Participant Characteristics

Twelve adults with PID, 24 to 65 years of age (median age 47 years), participated in the study. Eleven participants were ethnic Norwegian, and one was of Asian descent. The distribution of men and women was equal. Eleven participants had CVID and one had CN. The median age when receiving a PID diagnosis was 31 years (range: 6–53 years). Table 1 presents an overview of the participants’ pseudonyms, age, type of PID, number of years since they received their diagnosis, and their regular medical treatment.

**Table 1.** Participant Characteristics Including Pseudonyms.

Pseudonym	Approximated Age	PID Diagnosis	Number of Years Since the Diagnosis	Treatment
David (M)	60–70	CVID	>15	Hospital-based IVIG
Adam (M)	50–60	CVID	> 5	Hospital-based IVIG
Paul (M)	60–70	CVID	5–15	Home-based SCIG
John (M)	50–60	CVID	>15	Home-based SCIG
Allan (M)	40–50	CVID	<5	Home-based SCIG
Tom (M)	50–60	CVID	5–15	Home-based SCIG
Maria (W)	30–40	CVID	>15	Hospital-based IVIG
Eva (W)	20–30	CVID	5–15	Home-based SCIG
May (W)	50–60	CVID	5–15	Home-based SCIG
June (W)	30–40	CN	5–15	Other medication
Anne (W)	30–40	CVID	<5	Hospital-based IVIG
Lisa (W)	40–50	CVID	<5	Home-based SCIG

*Note.* PID = primary immunodeficiency disease; M = man; CVID = common variable immunodeficiency; IVIG = intravenous immunoglobulin; SCIG = subcutaneous immunoglobulin; W = woman; CN = chronic neutropenia.

### GI Distress Perception and Symptom Pattern

Most participants reported having GI distress for most of their lives; some had symptoms from early childhood. Not all participants were convinced that PID had caused their GI distress; these participants considered other diseases, genetics, or stress to have caused their symptoms.

**Figure 2.**
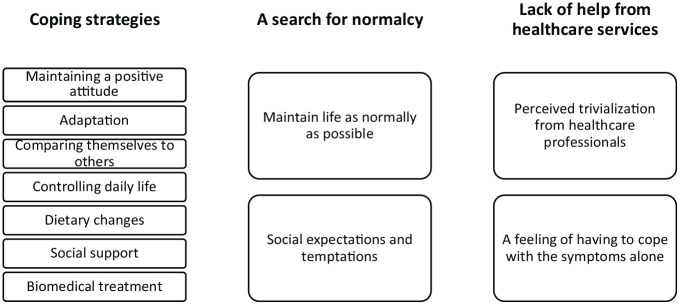
Overview of the Main Themes and Subthemes.

The majority of the participants were convinced of a relationship between intake of certain foods or beverages and GI distress. Still, their perceptions of the importance of diet as a treatment of GI distress differed. Some participants had never thought that a change in diet could relieve GI distress; others had tried to convince their physician about the effect of dietary modifications. More than one-half of the participants had experienced reduced GI distress after changing their diet. Many participants based their dietary modifications largely on personal experience without guidance from health care professionals. Most participants indicated that they had to take extra care in daily activities, such as social and physical activities and the proximity to a restroom, and diet to reduce GI distress. Overall, GI distress was described as periodic and highly unpredictable. Diarrhea was the most common symptom experienced and was frequently cited as the most difficult to cope with. Many described bloating and flatulence, and several participants stated that they felt bloated all the time. Some associated bloating and flatulence with physical discomfort and embarrassment, while others did not experience these symptoms as problematic. A minority experienced distressing abdominal pain and cramps. There were no clear trends based on the length of time with PID/years with diagnosis. The analysis gave rise to three main themes: *Coping strategies, A search for normalcy*, and *Lack of help from the health care system*. An overview of the main themes with subthemes is presented in Figure 2.

### Coping Strategies

All participants described the use of cognitive, behavioral, social, and biomedical coping strategies to manage their GI distress.

### Cognitive Coping Strategies

#### Maintaining a positive attitude

Many participants adopted a positive mental attitude that led them to feel in control. They chose to focus on the good periods and appreciated when they experienced only marginal GI distress. Some participants had developed an acceptance of the disease and a determination to make the best of the situation. Descriptions like “I cannot stop living because of it” and “I am seeing it as a part of life,” referring to GI distress, illustrate such a positive attitude. Others used a wait-and-watch-strategy, living one day at a time and not worrying about the future.

#### Adaptation

A recurring theme among the participants was adaptation; mastery of GI distress became a habit for them. Living with GI distress over the years had resulted in reduced attention to the condition, and the symptoms no longer bothered the participants to the same extent.

(. . .) there is a lot of loose stools. (. . .) Nevertheless, it does not matter to me. It still comes out, as it should. When you’ve had loose stools for over 10 years, it’s just a habit. (. . .) There is no problem there. I do not see it as a challenge, I do not. Not at all. (M)

There appeared to be a difference between men and women with regard to using adaptation as a coping strategy. While all male participants described adaption, only a few of the female participants mentioned adapting to GI distress.

#### Comparing themselves to others

Many of the participants stated that other people with PID were worse off than they were. Almost all the participants uttered this belief spontaneously. By performing this comparison, they found themselves to be quite fortunate.

There has been some focus on it [GI distress]. Because it is a problem that many people have or experience to different degrees. Again, when I hear what the others are saying, I understand that I am quite okay. Yes. (M)

Thus, the feeling of being fortunate in comparison with others is in accordance with the positive mental attitude that many participants had adapted.

### Behavioral Coping Strategies

#### Controlling daily life

Several participants found that daily life adjustments had improved their symptoms. Many felt they had the option to take control of their daily routines and find social support. While the male participants mainly seemed to choose adaptation to GI distress as their coping strategy, several female participants reflected on taking actions to improve their situation.

Well, it is like this . . . As an example, I like to hike in the forests and the mountains and so on. Therefore, I always bring paper with me. In addition, when I have to go to the toilet, I have to go in about 10 seconds, so I simply have to run into the forest. That is a bit annoying, but it does not stop me. And it does not prevent me from hiking. (W)

For both men and women, the unpredictable nature of GI distress was described as a challenge and as highly disabling, making the planning of everyday life difficult. Dietary restrictions or the need to maintain close proximity to toilets limited social life and activities.

Well, what I mostly take into consideration is the possibility of diarrhea. Because then . . . It’s not like you can wait for five minutes until you find a toilet. That’s not the way it is. No. Therefore, it is there and then. Yes. So it’s clear that I take considerations in that regard. (M)

The participants described attempts to control the symptoms by skipping meals, carefully calculating the timing of food intake in relation to daily travel, work, social events, or night-time activities.

#### Dietary changes

The majority of the participants had made some modifications to food and beverage intake in an attempt to minimize GI distress. They tended to base these modifications on personal experiences acquired over time.

I have sort of figured what I can tolerate and not tolerate. In addition, it’s mainly fatty foods, such as heavy foods and heavy sauces and such things, milky foods, and spicy foods that affect me. Therefore, I try to avoid that. (. . .) When I on a rare occasion have eaten porridge, I usually have to go straight to the bathroom. It’s almost before I finish eating. So it’s really quite easy to figure out [the connection between food and GI distress]. (W)

By avoiding food and beverages that triggered symptoms, many participants felt a sense of empowerment and being more in control of GI distress. Commonly mentioned trigger foods included baked goods such as buns and other refined wheat products, dairy products, foods rich in fat, spicy food, red meats, and sweets. The beverages mentioned most often as leading to symptoms were alcoholic drinks, soda, and acidic juices. After eating specific foods, the participants experienced discomfort like diarrhea, bloating, nausea, or gas formation.

However, the awareness of such triggers varied largely. One participant claimed he had never thought of the idea that specific foods could have an effect on his GI distress. He also had no experience of health care professionals raising diet as a topic.

No, there has never been any talk about any diet because of the immunodeficiency, so . . . No, I have not thought of it at all. It has never been on my mind that it would help. (M)

Several participants reported that they had not made any dietary adjustments, yet it emerged through the interviews that they actually avoided certain food items because they felt that these items made their GI distress more severe. Others had problems describing the actions taken because they “go automatically.” They expressed that their choices were made mainly due to habits rather than to a conscious effort to reduce GI distress. Participants avoided trigger food items automatically, without feeling that such avoidance was an extra consideration in everyday life.

The timing of meals was a frequently used behavioral coping strategy to maintain control to, for example, prevent night-time toilet visits. Several participants also practiced eating small, frequent meals instead of three or four large daily meals.

Of course even without thinking about it, you take caution. To what you eat, and not least when you eat. Eating in the evening is a no-go. Such simple things you really do not think about, but it just ends up that way. (M)

Some participants used special food items to reduce GI distress. They ate yogurts and drank probiotic milk in periods with symptom escalation, and they associated these food items with reduced diarrhea symptoms. Fruits and vegetables were also mainly associated with positive effects on GI distress, especially on constipation.

Although the majority of participants considered diet an important issue in controlling GI distress, they nonetheless described a relaxed and comfortable relationship with food and denied that diet restrictions led to stressful eating or impaired appetite.

Mirroring the coping strategy of maintaining a positive attitude, several participants expressed positive consequences from dietary changes. Most participants agreed that eating according to official nutrition recommendations was preferable in terms of reducing GI distress. Several described a growing interest in and enthusiasm for the influence nutrition had on their health after experiencing positive effects from their own dietary changes.

In that regard, I’m happy then, because I get more nutrients now than in two slices of bread and a cheese slice, right. Therefore, that has made me become a more conscious consumer. And I have transmitted that to the children (. . .) That’s very positive. (W)

Some participants reflected on the change in their taste preferences and the fact that foods causing them GI distress no longer tempted them in the same way.

So it’s really just nice that I cannot tolerate fatty foods. Now I’m not fond of it either. Because I’m not used to it. So it now opposes me a bit. (W)

Many said that without their GI distress, they would have engaged in less healthy eating, and they were thus grateful for a healthy change in diet.

### Social Coping Strategies

Participants expressed receiving benefits from exchanging experiences with people with similar disease histories. Support from people in the same situation was rewarding.

I have talked a lot with her [a friend]. She has had a very similar disease history as me. In addition, we had very much in common. Therefore, in that way, I recognized very much of what she said. So she has helped me a lot. (W)

Experiences from engaging in the Norwegian Patient Organization for Primary Immunodeficiency varied. Some felt the organization was a forum in which they had to accept an unwanted sick role. For others, the social support they received was crucial in their process of coping. Although a few participants described how people showed limited understanding for their dietary restrictions, the general view was that partners, family, and friends were supportive.

### Biomedical Coping Strategies

In general, participants believed that optimal medical treatment in terms of maintaining stable immunoglobulin levels was beneficial for their overall health. Some had experienced improvement in their GI symptoms after initiating Ig treatment, yet many others had experienced no change in their GI distress. Recommendations for biomedical treatment for GI distress in PID are limited. Some participants used over-the-counter medications to treat symptoms. Prophylactic use of antidiarrheal medications during work or before important social occasions was routine to several participants. The most commonly cited medication was loperamide hydrochloride. Some participants found probiotics in capsule form to be beneficial.

### A Search for Normalcy

#### Maintain life as normally as possible

A majority of participants wanted PID to play as small a role as possible in their everyday lives. One participant expressed her gratitude for receiving intravenous Ig treatment at the hospital instead of subcutaneous home treatment because she wanted to avoid a constant reminder of illness:

I’m getting ill, or mentally ill, of home treatment. I get all . . . When I open the fridge and see hundreds of glass bottles with stuff, I should take before Friday, or . . . “Oh shit, now it’s two days since last time, now I have to take it today,” and “Oh, it’s no time.” It’s never time, you know. And you get reminded that you are ill every time you open the fridge. (W)

Particularly in adolescence, this participant had experienced having PID as socially detrimental.

My mindset to this disease was that I tried to pretend it did not exist. Would not take medication, would not talk about it. You just want to be a normal fourteen-year-old that fits into the box with all the other fourteen-year-olds. So I took very little care, but I had many [GI] problems (. . .) So, I knew, that if I ate a bun or fine bread or something like that, then . . . But that [bun] was so important to me then. (W)

Nevertheless, it was important to several participants to state that PID and GI distress did not prevent them from living as they wanted. They perceived that PID or GI distress did not have an impact on everyday tasks.

Interviewer: Do you think it [PID diagnosis] affects your everyday life?

M: No. No. I will not let it affect it. In addition, I’ve grown up with . . . I’ve lived normally all the time. Actually. There has never been taken any special considerations for anything, ever.

A recurring theme was the fear of bothering other people in food-related contexts.

It’s so much extraordinary about me. So I do not want to be even more “special,” and not be invited by people because “No, we can’t ask her, because she only eats weird foods” and a little bit so on. (W)

Many felt that dietary restrictions made the disorder visible and revealed their condition to others. Special dietary needs created a perceived distance between themselves and others, challenging their desire to feel “normal.”

#### Social expectations and temptations

Despite the struggle of managing and controlling their GI distress, participants occasionally felt compelled to give in to dietary temptations. Factors such as taste and social meals weighed heavily when it came to giving in to temptations. There was a consensus among the participants that the biggest challenge was when others prepared the food. Some said that others expected them to eat “normally.” Most participants said that if others prepared the food, “then you eat it.” They felt that friends and family did not accept their dietary restrictions. Several of the participants expressed that they were afraid of negative responses in social situations. They found it easier to eat what people served them or to simply avoid eating rather than discussing their condition.

It’s hard when you’re at a dinner or party or something. Then you usually eat all day, snacking on different foods. I cannot do that. Then I get in trouble. (. . .) Therefore, it’s a bit like . . . It indeed makes you less social. It’s better to avoid going to parties and so on, instead of being there and everyone starts asking “Why don’t you eat anything? Are you not hungry?” Yes, that’s actually a restriction. (W)

The participants described how traveling, social settings, and everyday stress could make dietary modifications challenging. As one woman reflected: In an ideal world, she would have made all the beneficial choices to avoid GI distress; however, in real life, this was too demanding.

So in an ideal situation where you had eaten regularly, drinking a lot of water, eating the perfect foods, that would have been another situation, maybe. (W)

Others thought it was wrong to adjust the whole family’s diet or to prepare special dishes for themselves.

And starting to make a separate dinner only for me feels a little bit incorrect, in regard to the children when I say they should eat what we all eat. Yes. So then you have to find something like a golden mean. (W)

Thus, the fact that food and meals are important parts of social life made it challenging for many of the participants to make the choices they know could relieve their GI distress.

### Lack of Help From the Health Care System

#### Perceived trivialization from health care professionals

Only a minority of the participants had received in-depth information or a follow-up for GI distress related to PID from health service professionals. Some participants had experienced that physicians showed minimal interest regarding their GI distress and diet. This led to frustration.

Yes I told my physician, but he said, “Then you just keep away from fish, eggs, and milk.” Nothing else, they did not check anything, just said I should keep away from it. (M)

Lack of help led to negative thoughts for some individuals, who expressed a fear of the future and worrying about their symptoms.

#### A feeling of having to cope with the symptoms alone

The participants had the sensation of being helpless in trying to manage their symptoms alone. Many experienced that physicians told them that GI distress is “common” in a PID diagnosis. Some found this comforting, while others found it frustrating. A few participants had sought help from alternative health care providers.

When you are that ill, and you get ill and you do not get well, then you are willing to try everything. So, you eventually will become almost desperate. Because you don’t want to live that life [as ill]. (W)

A few participants had experienced disbelief on the part of health professionals when they mentioned their positive dietary experiences.

Yes, I have also received some feedback on . . . So when I say that I have reduced the number of yearly antibiotic cures from 11 to 4, the physicians say: “Oh, that’s awesome!” So I say “Yes, I’ve changed to a ‘most-of-the-time vegan’ diet.” Then they just say “Oh, okay” and they roll their eyes and think: “There’s probably something else.” Therefore, I feel they do not believe me. (W)

The lack of advice from their physicians or confusion and frustration regarding dietary precautions led several participants to seek information from alternative sources. The internet and social media were the most widely used sources of information.

## Discussion

This study revealed that the physical and psychosocial aspects of living as an adult with primary immune deficiency and GI distress are complex. It also revealed that participants strive to lead a normal life in spite of GI distress, and yet they feel that they must deal with GI distress by themselves, without help from the health care system.

Many participants claimed initially that GI distress did not affect their lives and that they did not take any dietary precautions. However, the actual impact of GI distress gradually emerged, and it became obvious that it indeed affected their everyday life. The participants seemed to experience GI distress as shameful and embarrassing to talk about, similar to findings in several other studies (Bjorkman et al., 2014; Farndale & Roberts, 2011; Rønnevig et al., 2009). GI symptoms are intimate and often connected to social norms about acceptable behavior, thereby threatening the dignity of sufferers (Rønnevig et al., 2009). In addition, some people perceive that GI distress is just “a part of life,” and as such, the threshold for seeking health care for these problems is often high (Drossman et al., 1993). Several studies have reported that despite the high prevalence of chronic or recurrent GI distress in the general population, few individuals actually consult health care professionals about their symptoms (Gaburri et al., 1989; Koloski et al., 2002; Sandler et al., 1984). In addition, the unclear etiology and lack of evidence-based treatment for GI complications in PID (Uzzan et al., 2016) may cause physicians to refrain from asking patients about GI distress. This leaves the patients to search for symptom relief and coping strategies on their own.

### Coping Strategies

This study revealed a wide and diverse range of coping strategies used to manage GI distress in individuals with PID. A common approach was the use of a positive mental attitude. This finding is in accordance with that of another Norwegian study on individuals with PID (Sigstad et al., 2005), which described an optimistic coping strategy as the most frequently used strategy for dealing with the disease. In that study, coping with the disease was related to the person’s sense of closeness, in terms of social networks, and competence, in terms of knowledge, skills, and experience of usefulness (Sigstad et al., 2005). In the present study, the social network was a source of both support and challenge. The general view among the participants was that partners, family, and friends were supportive, even though many described social gatherings as challenging.

Several participants appreciated their membership in the PID patient organization. Fellowship and peer meetings may be a source for support and knowledge. Other individuals, however, may avoid such companionship because of possible negative experiences (Garrino et al., 2015)—as described by some participants in our study, who felt that there was too much focus on the disease in peer meetings.

Our study revealed several strategies aimed at normalizing everyday life; for example, comparing oneself to people with more serious conditions, reluctance to acknowledging having the condition, or a wait-and-watch strategy. This coincides with strategies developed by people with rare disorders (von der Lippe et al., 2017).

Some strategies appear to be more effective than others are. The use of avoidance, distraction, and disengagement techniques are associated with lower quality of life among people living with rare genetic conditions. On the other hand, acceptance, optimism, and hopefulness are associated with higher quality of life (Cohen & Biesecker, 2010).

Although it was not a focus of this study to explore differences in how male and female individuals with PID adapted to the GI distress, the men and women did present their adaptation differently. The women talked more about emotions in relation to GI distress, as well as a lack of understanding from others. The men described that living with GI distress had become a habit, and they seemed less inclined to search for actions or remedies to relieve their symptoms. The female participants seemed, on other hand, to cope by searching for actions to relieve the symptoms. Experiences of living with irritable bowel syndrome (IBS) are different for men and women: Men demonstrate masculinity, whereas women are more concerned with relational responsibilities in line with societal roles and expectations (Bjorkman et al., 2014). Due to similar GI symptoms and lack of effective treatment, it is conceivable that these differences also apply to male and female individuals with PID and GI distress. However, to illuminate possible differences, we need more research.

Dietary changes were the most frequently used behavioral coping strategy among the participants. More than one-half of the participants had experienced reduced GI distress from dietary modifications. Through experimentation and over time, several participants had identified food items that triggered GI distress. There were striking similarities in the foods and beverages presented as triggers. The most common trigger foods mentioned were refined bread and baking products, dairy products, food items rich in fat, spicy food, red meat, and sweets. Among beverages, the participants mentioned alcoholic drinks, carbonated soft drinks, and acidic juices. Many of these foods and beverages overlap with triggers mentioned by people with IBS (Fletcher et al., 2008; Schneider et al., 2009; Skrastins & Fletcher, 2018). These triggers also overlap with foods containing higher quantities of FODMAPs (fermentable oligosaccharides, disaccharides, and polyols), which have been shown to trigger abdominal symptoms such as bloating, flatulence, abdominal discomfort, and diarrhea in, for example, IBS (Gibson & Shepherd, 2010).

### A Search for Normalcy

A further important finding was a desire to be “normal.” The participants did not want the disease to have any impact on their life. This is also described in other studies on individuals with rare chronic diseases (Garrino et al., 2015). Most participants were determined to live as normally as possible while attempting to manage their PID and GI distress.

Food and the sharing of meals are tied to strong normative rules. Being unable to eat the same foods as everyone else has social consequences and may explain why most participants refrained from following their personal diet regimens in social gatherings. Several studies have reported the challenges of maintaining dietary regimens (Diesen et al., 2015; Olsson et al., 2009). Thus, to accept foods that increase GI distress may “make sense” socially, as conveyed by the participant who described how she denied having the disease as a teenager. This shows that the stigma of adhering to a special diet can become unbearable in some social settings despite the possible negative health consequences of nonadherence.

A study of adolescents with celiac disease described eating special foods at social gatherings as a way of making an invisible condition visible, in addition to generating a feeling of guilt when refusing food offered by others (Olsson et al., 2009). This shows how expectations and norms related to food and meals may serve as an intrinsic barrier to dietary adherence. Many participants in the present study had initiated their dietary regimen on their own without advice from health care professionals. Nevertheless, some described dietary restrictions as an extra burden because of a lack of understanding in their social network as well as in the health care system. In addition, even if they had personal experience of their diet reducing GI distress, there are no known health consequences of deviating. Therefore, it is possible that people with PID find it even more difficult to receive social acceptance for their dietary changes. The participants’ attitudes toward dietary changes as a treatment for GI distress seem to be linked to their coping strategies. Participants who used cognitive coping strategies based on maintaining a positive attitude and taking control of daily life seemed to be open to dietary changes. In contrast, participants who used cognitive coping strategies based on denial did not consider dietary modifications as an option.

### Lack of Help From Health Care Service

A repetitive topic among participants was the perception that health professionals did not take their GI distress seriously. Several participants had requested advice for their GI distress, yet did not receive an explanation or adequate help. If health professionals or researchers do not address this topic, then evidence-based knowledge about the possible effects of dietary modifications on GI distress in individuals with PID will remain unknown, and these individuals will be forced to rely on personal trial and error as well as information from other sources.

With guidance and follow-up from a clinical nutritionist, the likelihood of establishing an individualized diet with regard to GI distress and achieving a healthy nutritional composition will be greater. To provide individuals with PID with a clearer understanding of their condition, and to successfully address their concerns, it is essential that health care professionals have adequate knowledge regarding GI distress in PID and pay attention to its psychosocial consequences as well as physiological symptoms. This will likely contribute to improving individuals’ self-care ability and decreasing the negative impact of GI distress on daily life.

It is important in the follow-up of individuals with PID to acknowledge the fact that a simple question about GI distress may be insufficient for clarifying GI problems related to PID. The problems associated with the challenges to finding the cause of GI distress in individuals with PID also demonstrate the importance of qualitative research in eliciting and describing these individuals’ experiences with symptoms, as well as with symptom relief.

### Strengths and Limitations

The fact that PID is rare made the recruitment process demanding and as such limited the sample size in this study. However, the use of two different sampling strategies contributed to increased sample variation. The online invitation required participation to be based on individual initiative, while the sampling strategy at Oslo University Hospital was based on health care professionals knowing the patients’ condition. Another limitation is that all participants except one were ethnic Norwegian. Due to this recruitment process, the study cannot speak to the actual prevalence of GI distress among individuals with PID or, equally important, to the severity of GI distress. However, the study did yield knowledge on how those with GI distress experience and deal with the condition and its symptoms.

A strength of the study was its inclusion of a wide age span (24–65), different subgroups of PID, variable treatments, and age when diagnosed. Another strength was its equal distribution of men and women, balancing the representation of males/females. The sex categorization was based on answers from a questionnaire given to the participants during the interview. The participants originated in various parts of the country. This increased the heterogeneity of the sample, and thus increased data credibility (Howitt, 2016). That said, no men under 40 years of age participated in the study, and it would have been advantageous to conduct member checking of the analysis. However, this was not done because the study was part of a thesis with time limitation. To enhance credibility, the interviewer verified her interpretations with the participants during the interviews by summarizing questions to ensure that her understanding corresponded to what the participants meant to communicate. We consider the fact that the interviewer did not have any knowledge about the characteristics of PID diagnosis to be a strength of the study. She had no prior insights into or knowledge about the diagnoses, how people with PID handle their condition, nor did she have previous knowledge about the challenges associated with GI distress. This proved beneficial in ensuring that the interviewer remained open-minded during data collection and reflexive, without false presumptions, about the possible findings, as well as probing the answers by asking follow-up questions and making sure she had understood the answers correctly. Katrine Kjenstadbakk Brede discussed all the interviews and analysis with the other authors who had knowledge about PID and were trained in qualitative research methods.

## Conclusion

This study highlighted the strengths and abilities of individuals with PIDs and GI distress with respect to managing their difficult situation. The participants strived to maintain a normal life as much as possible, in spite of the challenges of their condition. They used several coping strategies to relieve and control their GI distress in daily life, including cognitive, behavioral, social, and biomedical coping strategies. The majority of the participants had made some dietary changes in an attempt to minimize their GI distress. These changes tended to be based on personal experiences acquired over time and were rarely assisted by health care professionals. Modifications to food and beverage intake were at times found to be challenging due to variable responses to potential triggers, but also because the participants wanted to avoid attracting social attention or bothering others with special diets. The perceived effect of the actions the participants took to limit GI distress varied. Although some had found effective coping strategies, most participants were missing guidance and advice from health care professionals aimed at mitigating GI distress when given a PID diagnosis. Persons with PID can discuss nutrition, eating habits, and GI symptoms in patient group meetings organized by patient associations. Courses or information meetings on PID organized by health care professionals should focus on potential GI challenges. Furthermore, persons with PID should be encouraged to emphasize their GI challenges in appointments with their health care providers, and health care professionals should more effectively support these individuals in their struggle to identify and find explanations and solutions for GI distress.

## Supplemental Material

sj-pdf-1-qhr-10.1177_1049732320967908 – Supplemental material for Primary Immunodeficiency Diseases and Gastrointestinal Distress: Coping Strategies and Dietary Experiences to Relieve Symptoms Click here for additional data file. Supplemental material, sj-pdf-1-qhr-10.1177_1049732320967908 for Primary Immunodeficiency Diseases and Gastrointestinal Distress: Coping Strategies and Dietary Experiences to Relieve Symptoms by Katrine K. Brede, Margareta Wandel, Ingrid Wiig and Charlotte von der Lippe in Qualitative Health Research
