# Supplementary figures and images for: Primary Immunodeficiency Diseases and Gastrointestinal Distress: Coping Strategies and Dietary Experiences to Relieve Symptoms
Source: Qual Health Res. 2020 Nov 4;31(2):361–72. doi: 10.1177/1049732320967908 (PMC7750663; doi:10.1177/1049732320967908)

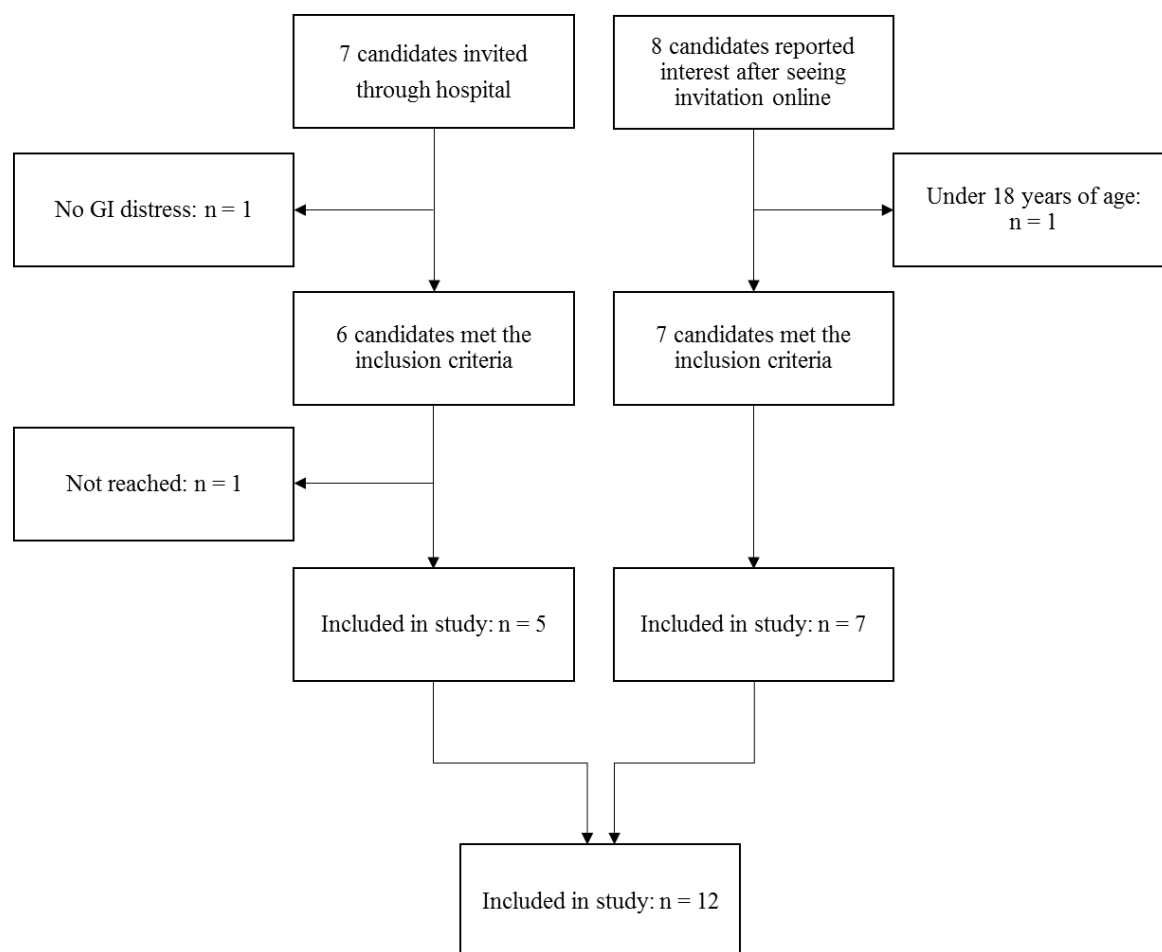

Figure 1. Recruitment process flowchart

Supplement: sj-pdf-1-qhr-10.1177_1049732320967908 – Supplemental material for Primary Immunodeficiency Diseases and Gastrointestinal Distress: Coping Strategies and Dietary Experiences to Relieve Symptoms [file sj-pdf-1-qhr-10.1177_1049732320967908.pdf]
